# Effect of Starch Types on the Textural and Rehydration Properties of Extruded Peanut Protein Pore Gel Particles

**DOI:** 10.3390/gels10040250

**Published:** 2024-04-07

**Authors:** Feng Guo, Anna Hu, Huan Zhou, Hui Hu, Tongqing Li, Qiang Wang, Jinchuang Zhang

**Affiliations:** Key Laboratory of Agro-Products Processing, Institute of Food Science and Technology, Chinese Academy of Agricultural Sciences, Ministry of Agriculture and Rural Affairs, Beijing 100193, China; 82101222184@caas.cn (F.G.);

**Keywords:** peanut protein, starch, extrusion puffing technology, gel particles, textural and rehydration properties

## Abstract

In this study, the effect of different starches from corn, potato and pea containing varying amylose/amylopectin ratios on the textural and rehydration properties of extruded peanut protein gel particles were investigated. Results showed that textural and rehydration properties of peanut protein extruded with corn starch, potato starch and amylopectin are slightly inferior to those of peanut protein with pea starch extrudates. The addition of pea starch led to an increase in the pore structure of the peanut protein extrudates and improved their water absorption index, simultaneously reducing the hardness and density. Pea starch, as a natural water-absorbing expansion material, helped peanut protein to form cross-linked gel polymers that bind more water molecules, in addition to further polymerization with peanut protein, which made the protein secondary structure became disordered. These changes directly affected the textural properties of the extrudates. In addition, the blended system of starches and peanut protein tended to form more elastic solids, which affected the expansion of the extrudates. These findings indicate that starch can effectively improve the poor expansion of proteins, making it suitable for use in the production of plant protein-based foods.

## 1. Introduction

Peanut (*Arachis hypogaea*) is one of the important oil crops. The by-products produced after oil extraction is called peanut meal, which, according to different protein extraction process, can be classified as peanut protein concentrate (protein content of 70%) and peanut protein isolate (protein content > 80%). Peanut protein contains 18 kinds of amino acids, including 8 essential amino acids that cannot be synthesized by the human body. It has a high digestion coefficient of up to 90% and is rich in anti-nutritional factors while having fewer flatulence factors [1,2,3,4]. Secondly, peanut protein is characterized by good solubility, gelation and other functional properties, which makes it have more application scenarios in the food processing field, such as peanut protein powder, peanut protein-based meat products, peanut protein milk drinks, functional short peptides and so on. Peanut protein is a characteristic agricultural resource that has not yet been fully utilized, with great potential for development. The development of new peanut protein products through modern food processing technology can enhance the added value of peanuts.

Extrusion puffing technology is a low-moisture extrusion technology (moisture content below 40%) with high energy utilization and great raw material suitability, which combines mixing, shearing, cooking, puffing and drying in an energy-efficient, fast and continuous process [5]. Starch and protein macromolecules form a mixed system under the action of thermal and mechanical energy in different heating zones, and ultimately the water molecules are vaporized at the extrusion die due to the drastic change in internal and external pressure difference, which transforms the polymer system into an expanded product with a more porous structure, accompanied by a crispy texture. In addition to this, extrusion puffing technology can improve digestibility and nutrient bioavailability in processed foods, which has led to increasing consumer interest in extruded snacks [6]. Previous studies have been conducted on starch-based extruded puffed products, and the nutrients of the products are relatively homogeneous. Recently, many studies have been conducted on the compounding of plant proteins with starch to produce protein-rich extruded snacks, and the effects of the interaction between proteins and starch in extrusion puffing on the textural properties of the puffed products, including hardness, brittleness, expansion ratio, density, and the rehydration properties, including water absorption index (WAI) and water solubility index (WSI) [7]. Lyu et al. [8] conducted low-moisture extrusion tests of soybean protein isolate, wheat protein and corn starch with different end-extrusion temperatures and found that the expansion ratio and rehydration properties of the extrudate increased with the increase in end temperature. Beck et al. [9] used pea protein combined with pea fiber and rice starch at 23% and 26% moisture content for extrusion puffing and found that different moisture contents had different effects on the expansion of the extrudates. Philipp et al. [10] investigated the effect of combining different pea protein contents with rice starch on the expansion of extrudates, and the results showed that pea protein isolate was added at a level of 10%, the expansion of extrudates increased by 60% compared to extruded rice starch alone, which had an expansion ratio of 4.1~5.0. As the pea protein isolate content increased to 30% and 50%, the expansion ratio of the extrudates decreased significantly, and the bulk density and hardness increased. It was shown that a suitable increase in protein content could enhance the expansion effect and textural properties of the extrudate. At present, studies about extrusion puffing effect on the starch-based gel particles are more explicit, while the effect of different starch types on the textural properties and rehydration properties of extruded plant protein gel particles is unclear, especially for the peanut proteins in which what role they play is little known.

In the extrusion process, starch and protein form hydrogen bonds with water molecules, starch and protein combine with electrostatic interactions, hydrophobic interactions and disulfide bonds to form a gel structure, and the water-absorbing expansion of the starch–protein structure is dominated by starch [11]. The gel structure is filled with water molecules, which provides sufficient steam power for the subsequent expansion, increases the expansion ratio of the extrudate, and is conducive to the formation of pore structure of extrudate as to changing its textural properties [12,13]. It has been shown that the pore structure has an influence on the rehydration of extruded puffed products, in which products with uniform pore distribution and thin pore wall thickness showed better rehydration properties. In addition, the density of the product can directly characterize the expansion effect, high-density products represent expansion difficulties, with dense pore structure and hard texture, which is not conducive to chewing [14]. The edge density of the product was much larger than the center density, indicating that extrusion led to more breakdown of the edge structure, and the high density of the edge may have an effect on the brittleness of the product, which needs to be further investigated [15].

In light of the distinct extrusion processing characteristics exhibited by various starches and peanut proteins, this study focused on investigating the textural properties and rehydration properties of peanut protein-based gel particles under the effect of different starch types through extrusion puffing technology. By analyzing the textural properties, macro and micro structures of the extrudates, this study elucidates the influence mechanism of starch on the functional properties of peanut protein-based gel particles. These findings would establish a solid theoretical foundation for further application of extrusion puffing technology in producing plant protein-based foods.

## 2. Results and Discussion

### 2.1. Thermal Properties of Starch

**Figure 1:**
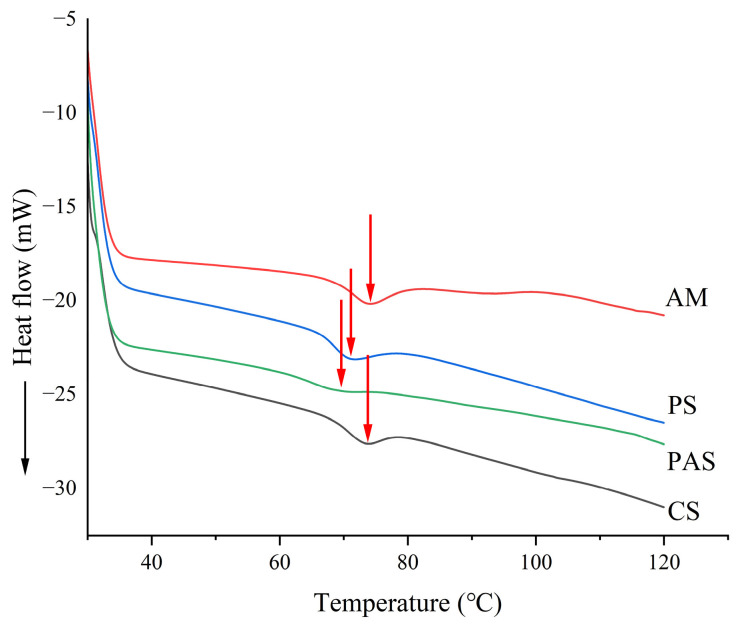
DSC of different starches (amylopectin, AM; potato starch, PS; pea starch, PAS; corn starch, CS). The arrows indicate the peak temperature of different starch.

The onset temperature, peak temperature, enthalpy change and peak width at half height of thermal transformations occurring in different types of starch were significantly different. As shown in Table 1 and Figure 1, the onset temperature of the thermal transformation of starch ranged from 61.89 to 68.02 °C. Pea starch had the lowest onset temperature while amylopectin had the highest, indicating that pea starch required less energy for thermal transformation. In addition, the enthalpy of thermal transformation of different kinds of starch ranged from 7.31 to 14.19 J/g, with amylopectin having the largest enthalpy and pea starch having the smallest. The peak width at half height of thermal transformations of different types of starches varied from 0.14 to 6.66 °C, and the peak width at half height of thermal transformations of corn starch were the largest, indicating that its rate of thermal transformation was the slowest, while pea starch was more prone to thermal transformation, and it was easier to polymerize with peanut proteins under the effect of thermal energy in extrusion puffing [16].

### 2.2. Expansion Property Analysis

**Figure 2:**
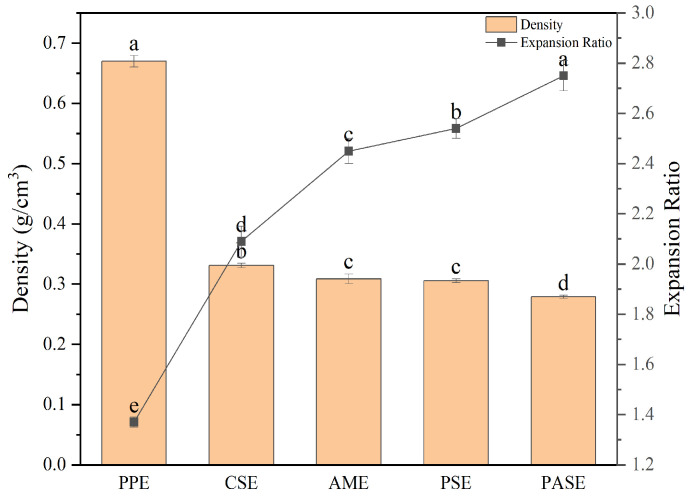
Expansion properties of extrudates of different starch and peanut protein extrudates (peanut protein extrudates, PPE; corn starch–peanut protein extrudates, CSE; amylopectin–peanut protein extrudates, AME; potato starch–peanut protein extrudates, PSE; pea starch–peanut protein extrudates, PASE). Different letters mean significant differences (*p* < 0.05).

Expansion properties can be described in terms of the extrudates’ expansion ratio and density. The expansion ratio of extrudates reflects the expansion effect of extrudates. A higher expansion ratio indicates a more significant expansion accompanied by a lower density [17]. The expansion ratio of PPE was significantly lower than that of the starch addition groups, as shown in Figure 2, suggesting that an excessive protein content inhibited extrudate expansion. The addition of starch to facilitate extrudate expansion may be attributed to the excellent water absorption properties of starch. During the extrusion process, the combination of water and starch enhanced the water absorption characteristics of the starch–peanut protein polymer, thereby improving its water storage capacity and providing robust steam power for expansion. Among them, the PASE exhibited the lowest density, indicating a pronounced puffing property of pea starch. This can be attributed to an optimal amylose/amylopectin ratio in pea starch, resulting in enhanced puffing effects of both pea starch and peanut protein [18].

### 2.3. Color and Textural Property Analysis

**Figure 3:**
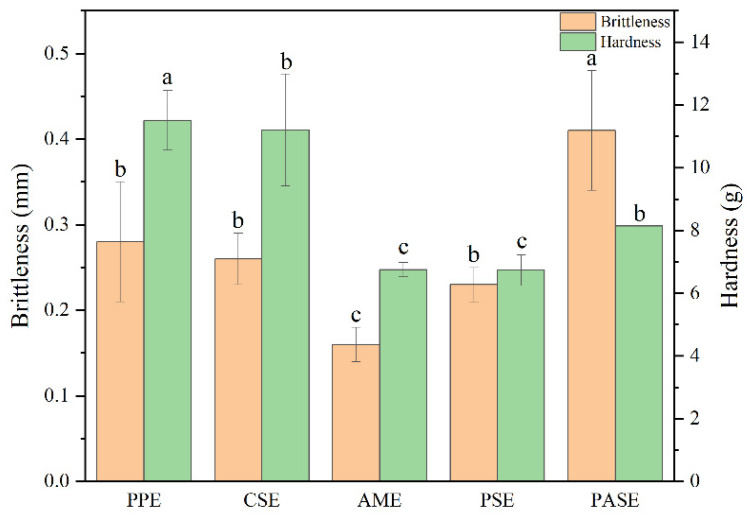
The brittleness and hardness of extrudates made from various types of starch and peanut protein (peanut protein extrudates, PPE; corn starch–peanut protein extrudates, CSE; amylopectin–peanut protein extrudates, AME; potato starch–peanut protein extrudates, PSE; pea starch–peanut protein extrudates, PASE). Different letters mean significant differences (*p* < 0.05).

For the color of PPE, it had the highest *a* value and Δ*E* value. The addition of starch increased the *L* value of extrudates, and pea starch improved the brightness of extrudates most obviously (Table 2). The hardness and brittleness of different extrudates are shown in Figure 3. Hardness is measured by destroying the mass (g) of the extrudates. The brittleness is expressed by the size of the fracture distance (mm) of the extrudates. Starch could significantly reduce the hardness of peanut protein extrudates, and the hardness of PASE, PSE and AME were reduced to 9 g. This indicated that starch can improve the palatability of the extrudates. Furthermore, the brightness of PASE was the largest at 0.41 mm. Poor brittleness generally showed that the fracture distance is larger. This finding was consistent with reports by other researchers that starch-protein mixed systems with higher protein content tended to form less brittle products at high temperature [19]. 

### 2.4. Rehydration Properties of Extrudates

**Figure 4:**
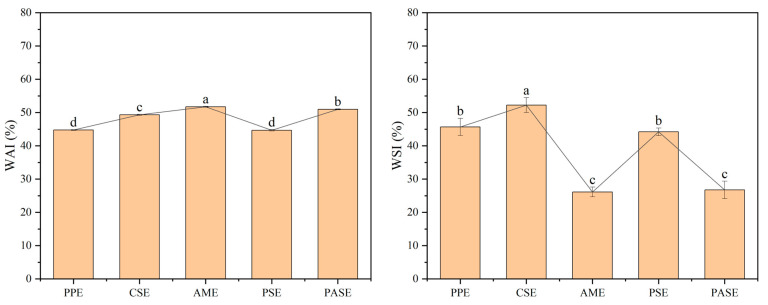
Rehydration properties of different starch and peanut protein extrudates (peanut protein extrudates, PPE; corn starch–peanut protein extrudates, CSE; amylopectin–peanut protein extrudates, AME; potato starch–peanut protein extrudates, PSE; pea starch–peanut protein extrudates, PASE). Different letters mean significant differences (*p* < 0.05).

The rehydration properties are described in terms of WAI and WSI for different extrudates. As shown in Figure 4, the WAI of the extrudate was roughly between 40% and 55%, and the addition of starch helped the peanut protein extrudate to absorb water better, which should depend mainly on the inter and intramolecular bonds induced between the protein and the gel polymers present in the starch due to the high temperature of the barrel, thus affecting the changes that the starch undergoes during the extrusion [20]. Additionally, the pore structure of the extrudate had an effect on its water absorption properties. The structure with dense pores and small pore diameters was not conducive to the absorption of water, while extrudates with uniform distribution of pores and small pore wall thickness absorbed water more easily [21]. This can be verified with the following analysis of the pore structure of the extrudates. Among them, AME had the highest WAI, indicating that its extrudate had the strongest water absorption capacity. It is possible that the amylopectin contained more hydrogen bonds, which interacted with water molecules and resulted in better water absorption of the extrudate. For the WSI of the extrudates, it was found that PASE and AME had higher WAI but lowest WSI, which were negatively correlated [22]. It showed that pea starch, amylopectin with protein extrudates were more soluble in water, probably because the large molecular aggregates were broken down or degraded to small molecules during expansion, which promoted the increase in WSI [23].

### 2.5. Macro and Micro Structures

**Figure 5:**
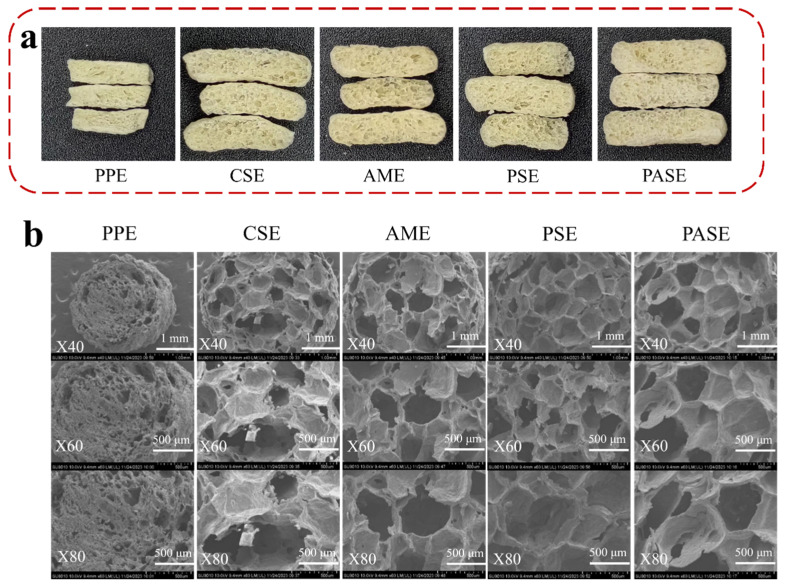
(**a**) Macro structure and (**b**) micro structure of different starch and peanut protein extrudates (peanut protein extrudates, PPE; corn starch–peanut protein extrudates, CSE; amylopectin–peanut protein extrudates, AME; potato starch–peanut protein extrudates, PSE; pea starch–peanut protein extrudates, PASE).

The macro pore structure of the extrudates resembled honeycomb and showed a random distribution. The addition of starch resulted in a loose pore structure for the extrudates, while also improving the dense pore structure of peanut protein extrudates (Figure 5a). The results of scanning electron microscopy (SEM) showed that the pore size of PASE was uniform and large, and the pore wall thickness was uniform and small (Figure 5b). The pore structure of PPE exhibited a relatively high density, with small pore sizes, potentially attributed to the tight binding between protein and water molecules, thereby impeding their extrusion. The pore structure of CSE, AME and PSE was relatively fragmented, with more cross-linked gel pores in the internal pore structure and uneven pore wall thickness, which was related to the brittleness of the extrudates. Thicker pore walls required greater mechanical force to break them, which was consistent with the brittleness results of the previous extrudates [24]. The results showed that the interaction between water and raw material during extrusion of different starch and peanut protein led to the intense degree of water vaporization during extrusion, and ultimately affected the pore structure.

### 2.6. Protein Secondary Structures

**Figure 6:**
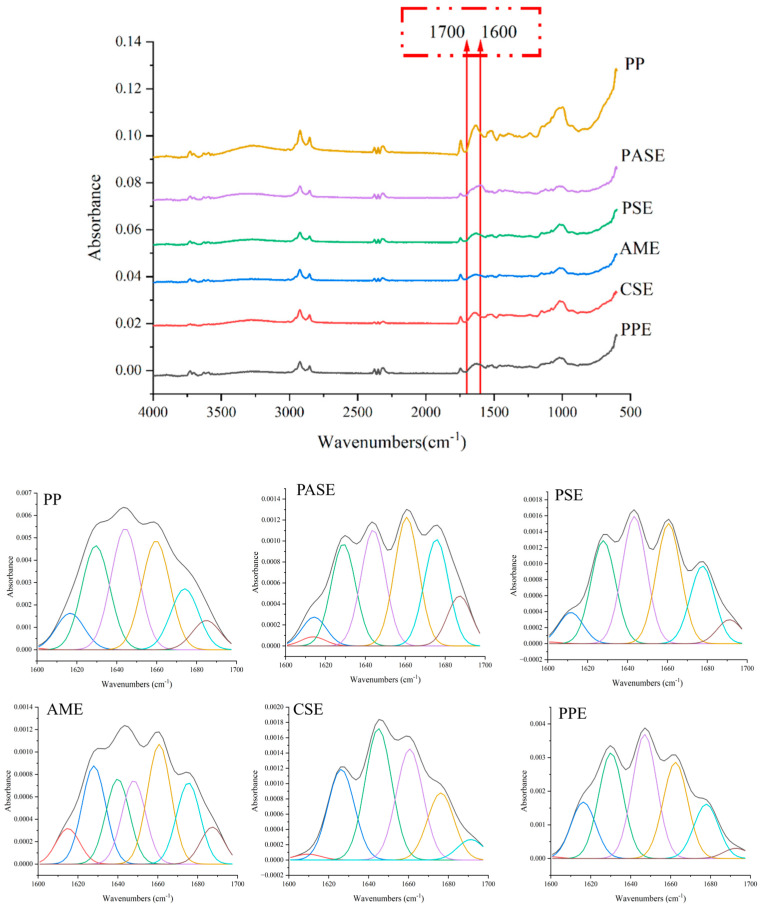
FTIR spectra of peanut protein and extrudates (peanut protein, PP; pea starch–peanut protein extrudates, PASE; potato starch–peanut protein extrudates, PSE; amylopectin–peanut protein extrudates, AME; corn starch–peanut protein extrudates, CSE; peanut protein extrudates, PPE).

The FTIR spectral characteristics and the relative content of secondary structures of protein are shown in Table 3 and Figure 6. Compared with the peanut protein raw material, extrusion puffing caused a significant decrease in the β-Sheet content, especially after the addition of starch, suggesting that starch bound tightly to the β-Sheet structure of the protein secondary structure and destroyed the β-Sheet during extrusion [25]. Therefore, starch connected groups in the β-Sheet structure to form macromolecules through hydrogen bonding, electrostatic interactions, and other forces during extrusion puffing, destroyed the original β-Sheet structure of proteins [26]. Peanut protein extrudates showed a decreasing trend in the content of Random coil structure. The addition of starch was favorable to combine with the ordered structure of protein, and destroyed the ordered structure leading to an increase in the disordered structure. The addition of amylopectin increased the content of α-helix structure in the extrudates, while the addition of the remaining starch did not significantly alter it. The results indicated that the increase in the α-helix contributed to the formation of more stable protein structures [27]. For the extrudates, the β-Turn content of the peanut protein extrudates increased, the addition of pea starch resulted in a similar increase in β-Turn [28].

### 2.7. Rheological Properties of Extrudates

**Figure 7:**
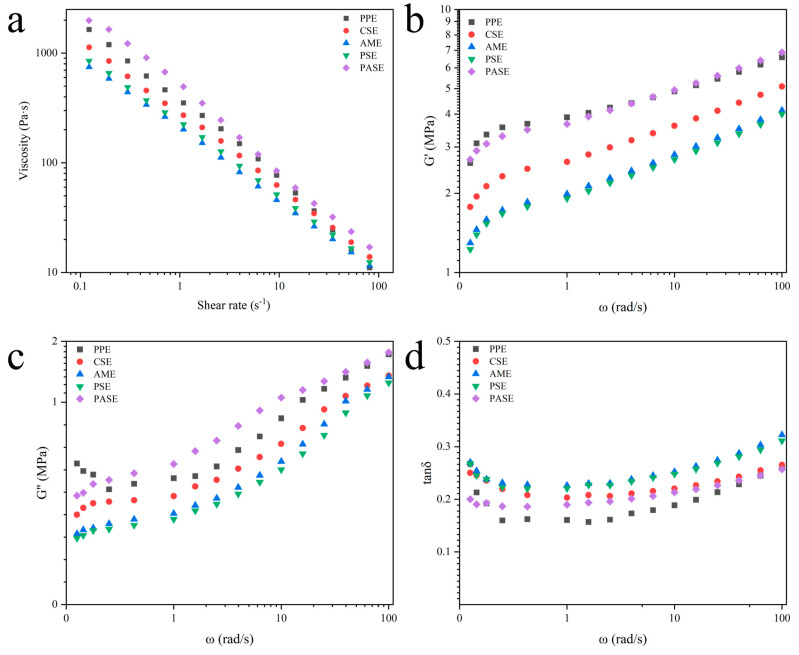
Rheological properties of different starch and peanut protein extrudates including (**a**) Viscosity, (**b**) G′, (**c**) G″, and (**d**) tan δ. (peanut protein extrudates, PPE; corn starch–peanut protein extrudates, CSE; amylopectin–peanut protein extrudates, AME; potato starch–peanut protein extrudates, PSE; pea starch–peanut protein extrudates, PASE).

It can be seen from Table 4 and Figure 7a that the flow behavior index (n) of the extrudates were less than 1. The extrudates showed a decreasing trend in viscosity with the increase in shear rate, which manifested itself as a non-Newtonian fluid. Possibly, the shearing action has caused a disruption of the intermolecular interactions of the extrudates [29]. Compared to PPE, the K value of PASE increased from 374.43 to 466.19, resulting in an increase in the viscosity of the mixtures, which was related to the fact that pea starch was susceptible to thermal transformation, and starch with a low thermal transformation temperature was more likely to produce an aggregated state with peanut proteins, which resulted in an increase in the viscosity of the mixture system. The significant decrease in viscosity and increase in mobility of AME may be attributed to the fact that the amylopectin’s bifurcated structure provided more binding surface area with peanut proteins and less spatial site resistance, resulting in lower viscosity, which was enhanced with shear, leading to the formation of a stronger mobility with peanut proteins. As shown in Figure 7b–d, G′ and G″ of the starch and peanut protein mixture system increased as the rise in angular frequency, which might be due to the fact that starch bound more water to increase the G′ of the solution through hydrogen bonding. The tan δ were less than 1, indicating that the samples tend to be more solid. This suggested that the starch interacted with the peanut proteins to form aggregates and increase elasticity.

## 3. Conclusions

The incorporation of starch in the extrusion process of peanut protein can significantly enhance the pore structure, reduce the hardness, and improve the rehydration properties of the extrudates. These changes can be attributed to the molecular interaction between starch and peanut protein during extrusion, which causes the absorption of water in the melt gel to generate more steam power, thus enhancing the expansion effect. In particular, pea starch exhibits a low thermal transition temperature, facilitating its polymerization with heated peanut protein. This interaction alters the secondary structure of proteins from ordered to disordered states while increasing polymer viscosity, thereby promoting extrusion puffing. These findings contribute to a comprehensive understanding of how starch modifies texture and rehydration properties in extruded products made from peanut protein, providing valuable technical insights for producing plant protein-based foods.

## 4. Materials and Methods

### 4.1. Materials

Peanut protein powder (purity > 60%) was supplied by Qingdao longevity Food Co., Ltd. (Qingdao, China). Corn starch (CS, purity > 90%) and potato starch (PS, purity > 90%) were provided by Leishi starch Co., Ltd. (Shenyang, China). Pea starch (PAS, purity > 90%) was obtained from Chuanfeng Food Co., Ltd. (Shandong, China). Amylopectin from Corn (AM, purity > 95%) was supplied by Shanghai Macklin Biochemical Technology Co., Ltd. (Shanghai, China). According to previous studies, the different amylose/amylopectin ratios of CS, PS, PAS and AM (PAS > CS > PS > AM) will affect the textural and rehydration properties of extrudates [11,30,31,32].

The thermal properties of starch were determined by differential scanning calorimetry (DSC), slightly modified based on a previous study [33]. DSC scans were conducted on a DSC-8000 thermal analyzer (PE, USA). A total of 3 mg of the sample was added to 9 μL of distilled water and placed in an aluminum pan. The moisture content of the sample was adjusted at 4 °C to make the water distribution in the sample uniform. Samples scanned from 20 to 120 °C at 10 °C/min in an inert atmosphere (50 mL/min of nitrogen). Peak temperature (T_p_), Onset temperature (T_0_), enthalpy changes (ΔH) and Peak width at half height (ΔT_1/2_) were calculated from the thermograms.

Peanut protein powder and starch were mixed at the ratio of 80:20 before extrusion. The material was stored at 4 °C for 12 h, so that the inside of the material reached a stable state.

### 4.2. Extrusion Puffing

The extrusion experiments were conducted using a twin-screw extruder (FMHE36-24, FUMACH, Changsha, China) with a screw diameter of 36 mm and a length–diameter ratio (L/D) of 24. The moisture content of blended ingredients was adjusted to 26% (dry basis) according to pre-experiments. The raw material was heated with five zones, set as Zone 1 = 60 °C, Zone 2 = 110 °C, Zone 3 = 130 °C, Zone 4 = 150 °C, and Zone 5 = 180 °C, respectively. The extruder feed rate was 26 kg/h, screw speed was 280 r/min and extrusion die diameter was 1.5 mm. The extruded particles (peanut protein extrudates, PPE; corn starch–peanut protein extrudates, CSE; amylopectin–peanut protein extrudates, AME; potato starch–peanut protein extrudates, PSE; pea starch–peanut protein extrudates, PASE) were dried by vacuum freeze-drying at −40 °C for 12 h to ensure the moisture content of the samples less than 7%. Then, the samples were freeze-dried at −40 °C about 12 h and then crushed to 80 mesh for subsequent analysis.

### 4.3. Expansion Properties of Extrudates

#### 4.3.1. Expansion Ratio (ER)

The expansion ratio of extrudates was calculated according to Equation (1). The diameter of the die attached to an extruder we used was 1.5 mm, and the cross-sectional diameter of extrudates was measured using a Vernier caliper (Shenhan Measuring Tools Co., Ltd., Shanghai, China) with five randomly selected samples.

(1) $$ER=\frac{Diameter\;of\;extrudate\;\left(mm\right)}{Diamter\;of\;extruder\;die\;\left(mm\right)}$$

#### 4.3.2. Density

The density of extrudates was measured following previous method of Hu et al. [34].

### 4.4. Textural Properties of Extrudates

#### 4.4.1. Surface Color Analysis

The extrudates were measured using a color spectrophotometer (CS-600, CHN Spec, Hangzhou, Zhejiang, China). Briefly, the extrudates were placed into a surface of a glass white plate. The color parameters defined as darkness/lightness (*L*), greenness/redness (*a*) and blueness/yellowness (*b*) were measured at least five times. The standard L*, a* and b* values of the calibration plate were 89.73, −0.78 and 1.88. The total color difference (∆*E*) of the extrudates was calculated by Equation (2) below:

(2) $$∆E=\sqrt{{\left(L-L^{*}\right)}^{2}+{\left(a-a^{*}\right)}^{2}+{\left(b-b^{*}\right)}^{2}}$$

#### 4.4.2. Hardness and Brittleness

Hardness and brittleness were measured with a TA.XT2 Texture Analyzer (Stable Micro Systems, Godalming, UK). The hardness and brittleness were determined by P/36R probe (cylinder, ∅36 mm). The test speeds were 2 mm/s (before test), 1 mm/s (in testing) and 2 mm/s (after test), respectively. The starting point force was 5 g, two compression interval time was 5.00 s and compression degree was 50%. Actually, the size of testing samples was approximately a cylinder with a diameter of about 2~4 mm and a length of 8~10 mm. It is placed directly on the test plate for hardness and brittleness measurement. Hardness is measured by destroying the mass (g) of the extrudes. The brittleness is expressed by the size of the fracture distance (mm) of the extrudes.

### 4.5. Water Absorption Index (WAI) and Water Solubility Index (WSI)

WAI was determined according to the method of Martin et al. [35]. Briefly, we weighed 1 g (*m*_0_) of extrudate powder into a centrifuge tube (*m*_1_), added 6 mL of distilled water, shook until the powder was completely dispersed, let it stood for 15 min to fully absorb water, then centrifuged at 4000 r/min for 15 min, discarded the supernatant, recorded the mass as *m*_2_, and then poured all the supernatant into a dry weighing bottle (*m*_3_). We dried it at 105 °C to a constant weight (*m*_4_). Each sample was measured in parallel 3 times and averaged. The water absorption index (WAI) and water solubility index (WSI) were calculated using Equations (3) and (4):

(3) $$WAI=\frac{m_{2}-m_{1}}{m_{0}}\times 100\%$$

(4) $$WSI=\frac{m_{4}-m_{3}}{m_{0}}\times 100\%$$

### 4.6. Analysis of Structure

A camera (D90, Nikon Corp., Tokyo, Japan) was used to assess the extrudates’ macrostructure and outlook. The extrudates were cut into a cylindrical shape measuring approximately 3 mm in diameter and 2 mm in height. Then, samples were dried in CO_2_. Dehydrated samples were coated with gold particles by a sputter coater (IB-5, Hitachi, Tokyo, Japan). Last, the microstructure of samples was observed by using a SU8010 instrument (SU8010, Hitachi, Tokyo, Japan) operating at an accelerating voltage of 10 kV.

### 4.7. Fourier-Transform Infrared Spectroscopy (FTIR)

FTIR analysis of the peanut protein powder and extrudates were measured using a spectrometer (TENSOR 27, Bruker, Bremen, Germany) on the surface of the Attenuated Total Reflectance (ATR) crystal. The spectral range was 500~4000 cm^−1^, the resolution was 4 cm^−1^ and the signal scanning was accumulated 64 times; each sample was measured 3 times. Peak fit 4.12 software (version 4.12, SPSS Inc., Chicago, IL, USA) was used to analyze the corresponding atlas of the amide band (between 1600 cm^−1^ and 1700 cm^−1^), identify the corresponding relationship between the location of each sub-peak and the secondary structure components, and obtain the proportion of the protein secondary structure. The content of protein secondary structure was determined by the absorption intensity of the amide region. The β-sheet was located at 1615~1637 cm^−1^ and 1682~1700 cm^−1^, the α-helix was at 1646~1664 cm^−1^, the β-turn was 1664~1681 cm^−1^, and the random coil was 1637~1645 cm^−1^ [21].

### 4.8. Rheological Properties

The rheological properties of extrudates are modified according to our previous research [36]. The test includes static cutting and frequency scanning. Distilled water was added to the sample to prepare a mixed solution with a concentration of 26%, which we covered with a sealing film and placed at 4 °C for water balance. A circular splint with a diameter of 40 mm was selected for testing, the distance between parallel plates was set to 1.05 mm, the excess samples were removed, and the samples were stabilized for 2 min. The test gap was set to 1 mm, the temperature was set to 25 °C, and the shear rate was set to 0.01~100 s^−1^. The fluid behavior of the solution was fitted by Power Law Equation (5):

(5) $$\tau =K\gamma^{n}$$

where *τ* is the shear stress (Pa), *K* is the consistency coefficient, *γ* is the shear rate (s^−1^), and *n* is the flow behavior index.

The frequency sweep test was performed at 25 °C with the angular frequency range of 0.1~100 rad/s at the linear viscoelastic region. The storage modulus (G′), loss modulus (G″), and the loss tangent (tan δ = G″/G′) were recorded.

### 4.9. Statistical Analysis

IBM SPSS 25.0 software (IBM Corporation, Armonk, NY, USA) was used for all analyses. Duncan multi-scope testing (*p* < 0.05) and single-way ANOVA were the next steps.

## Figures and Tables

**Table 1:** Thermal properties of starch (amylopectin, AM; potato starch, PS; pea starch, PAS; corn starch, CS). Different letters in the same column mean significant differences (*p* < 0.05).

Starch Types	T_0_ (°C)	T_P_ (°C)	ΔH (J/g)	ΔT_1/2_ (°C)
AM	68.02 ± 0.31 a	73.68 ± 0.28 a	14.19 ± 1.07 a	4.80 ± 4.66 b
PS	66.27 ± 0.01 b	70.87 ± 0.04 c	12.00 ± 0.09 b	0.58 ± 0.44 c
PAS	61.89 ± 0.44 c	68.18 ± 0.45 d	7.31 ± 0.10 c	0.14 ± 0.16 c
CS	67.00 ± 0.63 ab	72.13 ± 0.71 b	10.44 ± 0.33 b	6.66 ± 0.09 a

**Table 2:** Color changes of different starch and peanut protein extrudates (peanut protein extrudates, PPE; corn starch–peanut protein extrudates, CSE; amylopectin–peanut protein extrudates, AME; potato starch–peanut protein extrudates, PSE; pea starch–peanut protein extrudates, PASE). Different letters in the same row mean significant differences (*p <* 0.05).

Color	PPE	CSE	AME	PSE	PASE
*L*	57.04 ± 1.73 b	66.70 ± 0.58 ab	65.16 ± 0.18 b	66.38 ± 0.49 ab	67.69 ± 0.96 a
*a*	1.93 ± 0.11 a	0.40 ± 0.12 c	1.08 ± 0.31 b	0.72 ± 0.33 bc	0.53 ± 0.18 c
*b*	9.99 ± 0.57 b	10.81 ± 0.27 ab	11.89 ± 1.16 a	11.13 ± 0.62 ab	10.75 ± 0.90 ab
Δ*E*	33.79 ± 1.53 a	24.73 ± 0.63 c	26.61 ± 0.61 b	25.16 ± 0.68 bc	23.81 ± 0.55 c
Appearances	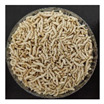	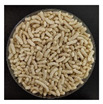	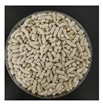	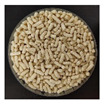	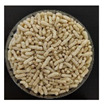

**Table 3:** The secondary structures (via FTIR) relative changes of peanut protein (peanut protein, PP; pea starch–peanut protein extrudates, PASE; potato starch–peanut protein extrudates, PSE; amylopectin–peanut protein extrudates, AME; corn starch–peanut protein extrudates, CSE; peanut protein extrudates, PPE). Different letters in the same column mean significant differences (*p <* 0.05).

Types	β-Sheet	Random Coil	α-Helix	β-Turn
PP	37.82 ± 1.82 a	25.51 ± 0.86 a	22.98 ± 0.67 b	13.29 ± 0.28 b
PASE	28.24 ± 0.20 b	19.84 ± 2.26 a	24.51 ± 1.03 b	22.03 ± 3.30 a
PSE	26.30 ± 0.41 b	25.43 ± 0.88 a	24.10 ± 0.71 b	16.24 ± 0.49 b
AME	27.61 ± 3.68 b	14.52 ± 1.74 a	39.39 ± 2.52 a	15.19 ± 0.20 b
CSE	27.54 ± 1.51 b	21.09 ± 10.56 a	26.05 ± 2.36 b	15.87 ± 2.59 b
PPE	32.45 ± 3.95 a	14.75 ± 1.24 a	26.37 ± 1.26 b	24.21 ± 1.58 a

**Table 4:** Power law equation fitting results of steady shear flow curves of different starch and peanut protein extrudates (peanut protein extrudates, PPE; corn starch–peanut protein extrudates, CSE; amylopectin–peanut protein extrudates, AME; potato starch–peanut protein extrudates, PSE; pea starch–peanut protein extrudates, PASE). Different letters in the same column mean significant differences (*p <* 0.05).

Extrudates	K (Pa·s^n^)	n	R^2^
PPE	373.4 ± 39.00 b	0.23 ± 0.02 b	0.99
CSE	292.47 ± 1.12 c	0.31 ± 0.00 a	0.98
AME	207.64 ± 6.75 d	0.35 ± 0.00 a	0.97
PAE	230.26 ± 1.06 d	0.34 ± 0.00 a	0.98
PASE	466.19 ± 28.16 a	0.26 ± 0.03 b	0.98

## Data Availability

The data presented in this study are openly available in article.
